# Overexpression of TRAF4 promotes lung cancer growth and EGFR‐dependent phosphorylation of ERK5

**DOI:** 10.1002/2211-5463.13458

**Published:** 2022-08-17

**Authors:** Siwei He, Danfeng Dong, Jiafei Lin, Beiying Wu, Xiaomeng Nie, Gang Cai

**Affiliations:** ^1^ Department of Laboratory Medicine Ruijin Hospital, Shanghai Jiao Tong University School of Medicine China; ^2^ Department of Respiratory Diseases Changhai Hospital, the Second Military Medical University Shanghai China

**Keywords:** epidermal growth factor receptor, extracellular signal‐regulated kinase 5, mitogen‐activated protein kinase, non‐small cell lung carcinoma, tumor necrosis factor receptor‐associated factor 4

## Abstract

Tumor necrosis factor receptor‐associated factor 4 (TRAF4) is overexpressed in a variety of carcinomas of different origins, but its role in tumorigenesis remains incompletely understood. Previous studies suggest that TRAF4 promotes epidermal growth factor receptor (EGFR) activation in non‐small cell lung cancer (NSCLC). However, the downstream signaling pathway of TRAF4‐mediated EGFR activation, as well as its effects on tumor cells, have not been fully elucidated. Here we report that TRAF4 overexpression is associated with increased activity of extracellular signal‐regulated kinase 5 (ERK5) in NSCLC tissues. Activation of ERK5 was dependent on TRAF4‐mediated EGFR activation, since inhibition of either TRAF4 or EGFR dramatically abolished phosphorylation of ERK5. Mechanistically, EGFR recruited mitogen‐activated protein kinase kinase kinase 3 (MEKK3), an upstream kinase of ERK5, in a TRAF4‐dependent manner. Thus, our data suggest that an EGFR‐TRAF4‐MEKK3‐ERK5 axis promotes the proliferation of tumor cells, and this may be a potential target for therapeutic intervention of NSCLC.

## Graphical abstract

In this study, an EGFR‐TRAF4‐MEKK3‐ERK5 axis in tumor cells was revealed to promote the uncontrollable proliferation of tumor cells. This pathway was activated in non‐small cell lung cancer tissues, but not in normal lung tissues.

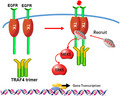

**Abbreviations**
BMK1: big mitogen‐activated kinase 1
EGFR: epidermal growth factor receptor
ERK5: extracellular signal‐regulated kinase 5
MAP3K3: mitogen‐activated protein kinase kinase kinase 3
MAPK: mitogen‐activated protein kinase
MEKK3: mitogen‐activated protein kinase kinase kinase 3
NSCLC: non‐small cell lung cancer
SCLC: small‐cell lung carcinoma
TNF: tumor necrosis factor
TRAF4: tumor necrosis factor receptor‐associated factor 4

Lung cancer is one of the leading causes of death in adult men and women [1]. Broadly, malignant lung tumors can be subdivided into small‐cell lung carcinoma (SCLC) and non‐SCLC (NSCLC), with adenocarcinoma most common in histology [1]. Although advanced molecular biology techniques have greatly accelerated the understanding of biological mechanisms underlying lung cancer development, the 5‐year survival rate has hardly improved in the last 50 years [2]. To understand the molecular alterations involved in cancer development, it is therefore essential to take the heterogeneity of the tumor into account. This is a prerequisite for a precise prognosis assessment and an ultimately adapted therapeutic strategy. A subset of genes was found overexpressed in cancers. Indeed, overexpression is one of the mechanism threads to the activation of proto‐oncogenes to oncogenes [3]. The tumor necrosis factor (TNF) receptor associated factor 4 (TRAF4) gene was identified due to its high expression in breast cancers [4]. Thereafter, the protein was found to be highly expressed in a variety of tumor tissues, including NSCLC [5]. TRAF4 was shown to be involved in signal transduction, as its TRAF structural domain mediates direct or indirect interactions with transmembrane receptors [6]. Additionally, TRAF4 possess E3‐ligase activity mediates poly‐ubiquitination of target proteins and leads to their activation or degradation [7]. However, the signaling pathway and the precise implication of TRAF4 in cancer are still unknown.

It has been widely found that epidermal growth factor receptor (EGFR) signaling is deregulated in NSCLC, and EGFR activation was demonstrated to be associated with multiple biological responses in tumor cells, including driving uncontrolled proliferation, conferring evasion of programmed cell death, and enhancing migration and metastasis [1, 8, 9]. Recently, TRAF4‐binding sites have been found in the intracellular structural domain of EGFR, and TRAF4 promotes phosphorylation of EGFR through regulating asymmetric dimerization of EGFR [10]. The relationship between EGFR/TRAF4 signaling and tumor proliferation was established [11] based on experiments with tumor cell lines. However, whether the downstream signaling cascades of TRAF4 mediate the activation of EGFR or not is still unknown. Moreover, what the role of the EGFR/GRAF4 signaling cascade is in clinical samples and its possible clinical significance remain unclear.

Extracellular signal‐regulated kinase 5 (ERK5), also known as big mitogen‐activated kinase 1 (BMK1), is a nonredundant mitogen‐activated protein kinase (MAPK) required for the maintenance of vascular integrity during development and tumorigenesis [12, 13]. ERK5 possesses an N‐terminal kinase domain that is highly homologous to ERK1/2 and a unique long C‐terminal domain. ERK5 activation is increased mainly in response to mitogens and oxidative and osmotic stresses upon phosphorylation by a MAPK/ERK kinase 5 (MEK5) [14]. MEK5 specifically recognizes the TEY motif present in the N‐terminal half of the protein within the catalytic domain, and is activated by mitogen‐activated protein kinase kinase kinase 3 (MAP3K3, MEKK3) and MEKK2 [15]. Compared with other members of the MAPK family, ERK5 is the least investigated one. In this study we found that the expression level of TRAF4 was positively related to the levels of active EGFR and phosphorylated ERK5 in NSCLC tissues. Mechanically, EGFR interacts with MEKK3 in a TRAF4‐dependent manner, which then triggers phosphorylation of ERK5. Since both EGFR and ERK5 are broadly expressed in various kinds of tissues, overexpression of TRAF4 in tumor cells leads to uncontrolled proliferation of tumor cells and development of NSCLC through setting up the EGFR‐TRAF4‐MEKK3‐ERK5 signaling axis.

## Materials and methods

### Mice and tumor monitoring

For the spontaneous growth assay, 2 × 10^6^ cells (single clones of *Traf4* ^−/−^ A549 cells) were injected subcutaneously into the back of 6–8‐week‐old NSG (NOD.Cg‐*Prkdc* ^*scid*^ *Il2rg* ^*tm1Wjl*^/Szj) mice in 1:1 Matrigel plus PBS. The growth of the primary tumor upon an orthotopic injection was examined by weekly measurements. Approximate tumor volumes were calculated by the formula 4/3 × 3.14 ×[(long diameter/2) (short diameter/2)^2^]. For the MEK5 inhibitor injection experiment, mice were injected with PBS, Bix 02189 (dissolved in DMSO, and added to PBS to a 5‐μm final concentration) alone every 2 days.

All animals were maintained in the Laboratory Animal Center at the Ruijin Hospital, Shanghai Jiao Tong University School of Medicine. All animal experiments were performed under the policies on using laboratory animals and were approved by the Animal Research Committee of the office for the Protection of Research Subjects at the University.

### Patient selection

We retrospectively reviewed the records of all patients who underwent surgery for lung adenocarcinoma at Ruijin Hospital and Changhai hospital from January 2008 to May 2015. All patients' tumor, node, and metastases staging were identified according to the American Joint Committee in Cancer guidelines, 7th edition [16]. Tumor specimens were collected during surgery to confirm the exact pathologic classification, tumor differentiation, and pathological tumor node metastasis (pTNM) stage. Patients who met the following criteria were considered eligible for inclusion in the study: 18 years of age or older; received surgery to remove the lesions completely; histologically confirmed without activating EGFR‐mutated NSCLC in stage IIIa; Eastern Cooperative Oncology Group (ECOG) performance status of 0 or 1; and adequate hematological, biochemical, and organ functions. Other exclusion criteria included: other malignancies before or during the study; any unstable illness; and pregnancy or lactation. This study was approved by the Medical Ethics Committee of the two hospitals. All experiments conformed to the Declaration of Helsinki, and written informed consent was obtained from each subject. More information of patients is shown in Table 1.

**Table 1:** Clinical characteristics of each group.

.Characteristic	*n*	Ratio (%)
Gender		
Male	26	78.8
Female	7	21.2
Age (year)		
< 60	20	60.6
≥ 60	13	39.4
Stage IIIa		
T4N0/T3‐4N1	16	48.5
T1‐3N2	17	51.5
Performance status		
0	25	75.8
1	8	24.2
Differentiation		
Poor	8	24.2
Moderated	13	39.4
High	12	36.4

### Cell cultures and transfections

The 293T, A549, and HeLa cells were grown in Dulbecco's modified Eagle's medium (DMEM), supplemented with glutamine, penicillin–streptomycin, and 10% FBS. Plasmids or siRNA were transfected using lipofectamin2000 according to the manufacturer's instructions (Invitrogen, Carlsbad, CA, USA). The cell lines were obtained from the Shanghai Cell Bank of the Chinese Academy of Sciences and stored in the laboratory.

EGFR and TRAF4 knockout cell lines were generated using the CRISPR/Cas9 technique. The gRNA sequences were derived from the GeCKO (v2) library (EGFR, 5′‐CCAATGAACGTTTGTGCGTC‐3′ and 5′‐CCTGACGCACAAACGTTCAT‐3′; TRAF4, 5′‐TGGGCCACTACGTCATCTAC‐3′ and 5′‐AGCCACAAAACTCGCACTTG‐3′), and were cloned into LentiCRISPR V2. For each gene, two constructs were used as a pool to improve the knockout efficiency in HeLa cells.

To construct the TRAF4 inducible cell line, the myc‐tagged TRAF4 cDNA sequence was cloned into the pTre3G vector. This vector was cotransfected with a p‐Max vector into Tet‐on HeLa cells. A single clone was chosen and identified by puromycin selection.

Cell growth was monitored by CCK8 assay according to the manufacturer's instructions (Sigma‐Aldrich, St. Louis, MO, USA). A total of 5 × 10^3^ cells were seeded in each well with a 96‐well plate on day 0.

### Antibodies and reagents

Tris, NaCl, and SDS for molecular biology and buffer preparation were purchased from Sigma‐Aldrich. Cell culture media and supplements were from Invitrogen (La Jolla, CA, USA). Antibodies were purchased from commercial sources as follows: anti‐EGFR (IHC‐00005; Bethyl Laboratories, Montigny, TX, USA), anti‐phospho‐EGFR [Tyr1068, Tyr992, and Tyr845 (Cell Signaling, Danvers, MA, USA)], anti‐TRAF4 (sc‐1920; Santa Cruz Biotechnology, Santa Cruz, CA, USA), mouse anti‐HA (H9658; Sigma‐Aldrich), Rabbit anti‐HA (Cell Signaling), mouse anti‐Omni and rabbit anti‐Omni (Santa Cruz Biotechnology), rabbit anti‐myc tag (Cell Signaling), mouse anti‐V5 antibody (Invitrogen), anti‐phospho‐ERK5 (Cell Signaling), anti‐ERK5 (Cell Signaling), anti‐phospho‐ERK1,2 (Santa Cruz Biotechnology), anti‐ERK1,2 (Cell Signaling), anti‐AKT (Cell Signaling), anti‐phospho‐AKT (Ser473; Cell Signaling), anti‐β‐actin (Cell Signaling). Recombinant human EGF was purchased from R&D (Minneapolis, MN, USA). Doxycycline and DMSO were purchased from Sigma‐Aldrich.

### cDNA construction

Human TRAF4 cDNA was cloned into N‐terminally tagged X‐press vector and C‐terminally tagged pcDNA3.1 vector. Human EGFR cDNA was cloned into the C‐terminally tagged HA or V5 pcDNA3.1 vector. Deletion or site mutants of TRAF4 and EGFR were made following standard PCR procedures.

### Western blotting and immunoprecipitation

All samples were denatured in a 1 × sample buffer [50 mm Tris–HCl (pH 6.8), 2% SDS, 2‐mercaptoethanol, 10% glycerol, and 1% bromophenol blue] for 5 min at 100 °C. Cells were lysed in a radioimmunoprecipitation assay (RIAP) buffer composed of 25 mm Tris–HCl (pH 8.0), 150 mm NaCl, 1 mm EDTA, 1 mm dithiothreitol (DTT), 0.1% SDS, 1% Nonidet P‐40, and 0.5% sodium deoxycholate. To analyze immune complexes, cells were lysed in a binding buffer containing 25 mm Tris–HCl (pH 8.0), 150 mm NaCl, 1 mm EDTA, and 0.5% Nonidet P‐40 for coimmunoprecipitation assays. The cell lysates were centrifuged (10,000 ***g***) at 4 °C for 5 min. All lysis buffers in this study contained proteinase and phosphatase inhibitors (Roche, Basel, Switzerland). Soluble fractions were precleared using protein G‐Sepharose at 4 °C for 15 min. Precleared cell lysates were immunoprecipitated for 1–4 h with the indicated antibodies. Immunocomplexes were adsorbed to the protein G‐Sepharose and were eluted by boiling for 5 min after three washes. Omni‐tagged proteins were immunoprecipitated with anti‐Omni. For quantification, fujifilm multi‐gauge V3.0 software (FUJIFILM, Tokyo, Japan) was used.

### Proximity ligation (Duolink) assay

For Duolink staining, pcDNA‐EGFR‐HA, pcDNA‐MEKK3‐M2, or pcDNA‐MEKK2‐M2 cotransfected TRAF4 inducible cells were treated with doxycycline or DMSO. After fixation and permeation, mouse anti‐HA and rabbit anti‐M2 antibodies were used to probe EGFR‐HA and MEKK3‐M2 (or MEKK2‐M2), respectively. Then, DNA labeled anti‐mouse IgG and anti‐rabbit IgG were used and followed by ligation reaction and amplification reaction as per the manufacturer's instruction (Sigma‐Aldrich). After DAPI staining of nuclear, the positive signal was captured with a Leica Confocal Microscope (Leica, Wetzlar, Germany).

### Tissue immunohistochemistry staining

Immunohistochemistry (IHC) staining was performed as described previously [17]. Briefly, tissue slides were serially deparaffinized, rehydrated, and subjected to antigen retrieval. The slides were then incubated with antibodies to target antigens and detected using the UltraSensitiveTM SP IHC Kit (Songkon, Shanghai, China), and then counterstained with hematoxylin. Two pathologists independently evaluated the staining. The protein levels of TRAF4 or Ki67 in the examined cases were classified as negative, low, intermediate, and high, according to both staining intensity and percentage of positively stained cells.

For immunofluorescence staining, cells were fixed in 4% paraformaldehyde and permeabilized in 0.5% Triton X‐100 for 30 min. Fixed cells were incubated with indicated antibodies overnight at 4 °C, followed by incubation with fluorescent‐labeled secondary antibodies. Nuclei were stained with 4′6‐diamidino‐2‐phenylindole (DAPI). Samples were viewed with a confocal fluorescence microscope system (NIKON Cl^si^; NIKON Instruments, Tokyo, Japan).

### BrdU staining

Cells were incubated with 10 μm BrdU (BD Pharmingen, San Diego, CA, USA) at 37 °C for 4 h, then fixed with 4% PFA for 15 min at room temperature. Fixed cells were then washed with PBS (3 times at 2 min for each wash) and incubated with 1 N HCl on ice (10 min), 2 N HCl at room temperature (10 min), and 2 N HCl at 37 °C (20 min). A borate sodium buffer (0.1 m, pH 8.5) was used for neutralization for 10 min at room temperature after removing the HCl solution. Cells were then washed with PBS (3 times at 2 min for each wash), blocked with 2% goat serum (with 0.3% Triton X‐100 in PBS, pH7.4) for 2 h at room temperature, and further incubated with rat anti‐BrdU primary antibodies (1:150; Santa Cruz, sc‐56,258) and Donkey anti‐Rat Alexa Fluor 488 secondary antibodies (1:200; Thermo Fisher Scientific, Waltham, MA, USA).

### RNA isolation and RT‐PCR

For RNA isolation, samples were thawed, minced with scissors, and homogenized using an OMNI TH tissue homogenizer (Omni International, Kennesaw, GA, USA). Homogenized samples were centrifuged at 10,000 ***g*** for 10 min, and the supernatant was used for RNA isolation from TRIzol (Invitrogen) according to the manufacturer's instructions. One microgram RNA from TPA‐treated skin samples was used to synthesize cDNA using Superscript II. Gene expression analysis was determined using the SYBR Greenmaster mix. CYBR Green PCR Master Mix (TaKaRa, Tokyo, Japan) was used for RT‐PCR. Primer sequences for RT‐PCR are as described before [17].

### Statistical methods

All values are presented as the mean ± SEM, unless otherwise indicated. Statistical significance was determined by a Student's *t* test or a one‐way ANOVA using graphpad prism 5.0 software (GraphPad Software, San Diego, CA, USA). A *P* value of less than 0.05 was used as the criterion for statistical significance.

## Results

### Expression of TRAF4 is significantly associated with poor prognosis in patients with NSCLC

**Fig. 1:**
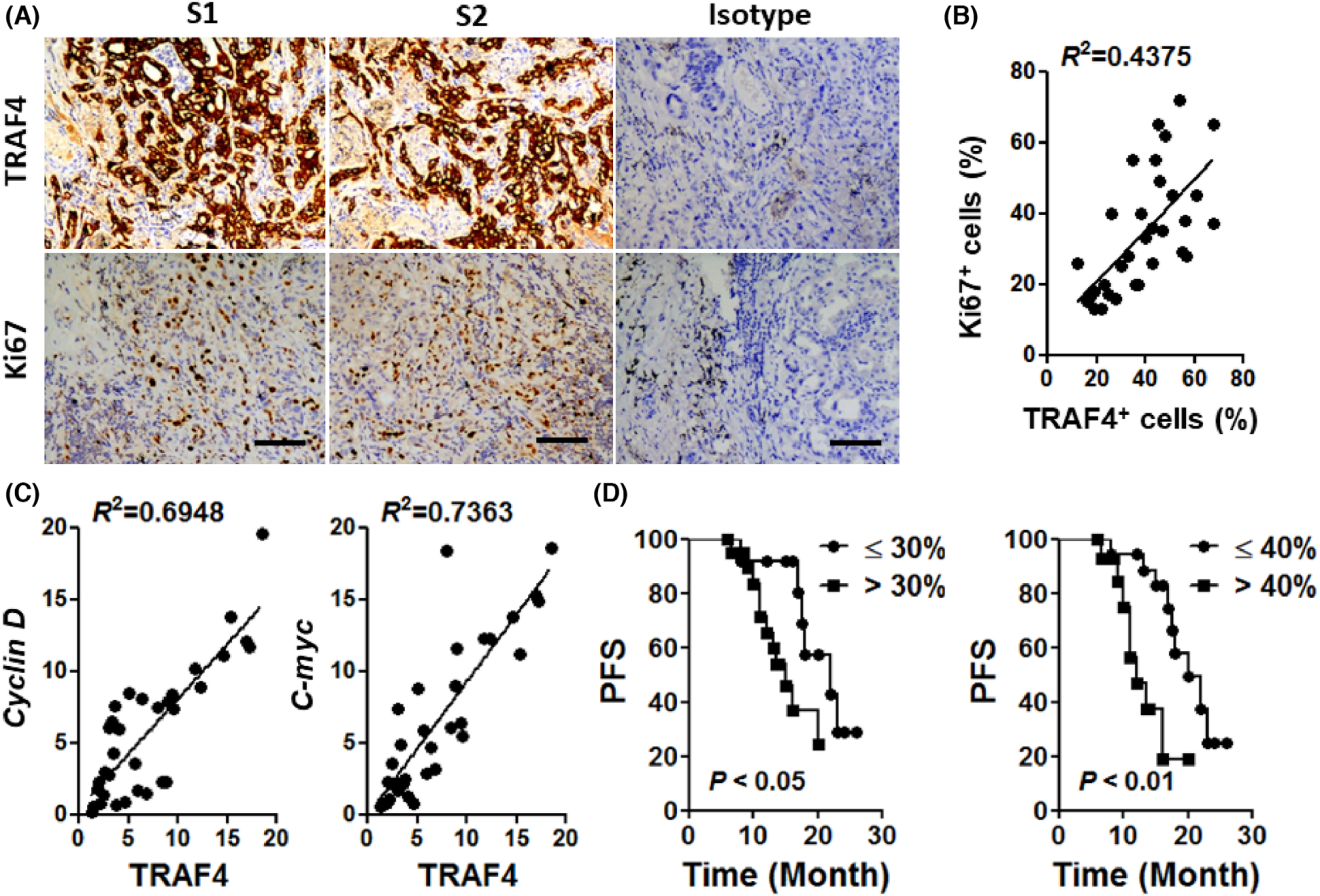
Higher expression of TRAF4 is associated with the progression of NSCLC. (A) Representative immunohistochemical staining of TRAF4 and Ki67 on lung cancer tissue from two patients with NSCLC. Scale bar, 50 μm. (B) The relationship between TRAF4 level and Ki67 level in lung cancer tissue from 33 patients with NSCLC. The protein expression level was evaluated by two pathologists of lung cancer. (C) Linear regression of the indicated mRNA level in human NSCLC tissues as detected by RT‐PCR. (D) The expression levels of TRAF4 are strongly correlated with PFS. Using 30% (left) or 40% (right) as the cutoff value of the TRAF4 level, patients with high TRAF4 expression had shorter PFS. R squared represents the coefficient of determination of the linear regression.

It has been found that the expression of TRAF4 is higher in NSCLC [5], but its impact on tumorigenesis remains largely unknown. To investigate whether TRAF4 plays a role in tumor proliferation, we first performed IHC staining of TRAF4 and Ki67 in 32 tumor tissues from patients with NSCLC of phase IIIa. The levels of TRAF4 and Ki67 were determined by both the staining intensity and the percentage of positively stained cells (Fig. 1A). We found that tissues with a higher level of TRAF4 protein also showed a higher Ki67 level in both quantity and intensity (Fig. 1B). Additionally, the mRNA level of *Traf4* was positively correlated with two characteristic genes of cell proliferation [18, 19], *Cyclin D* and *C‐myc* (Fig. 1C), suggesting that TRAF4 may play a critical role in tumor expansion. To further evaluate the impact of TRAF4 expression on the prognosis of lung cancer patients, we retrospectively studied the survival time of these patients, and found that a higher expression level of TRAF4 was associated with shorter progression‐free survival (PFS) (Fig. 1D). Additionally, we observed that patients with a higher expression level of TRAF4 also had a shorter overall survival (OS) time, although the difference between the two groups did not reach significance (Fig. S1).

### TRAF4 regulates cancer cell proliferation *in vivo*

**Fig. 2:**
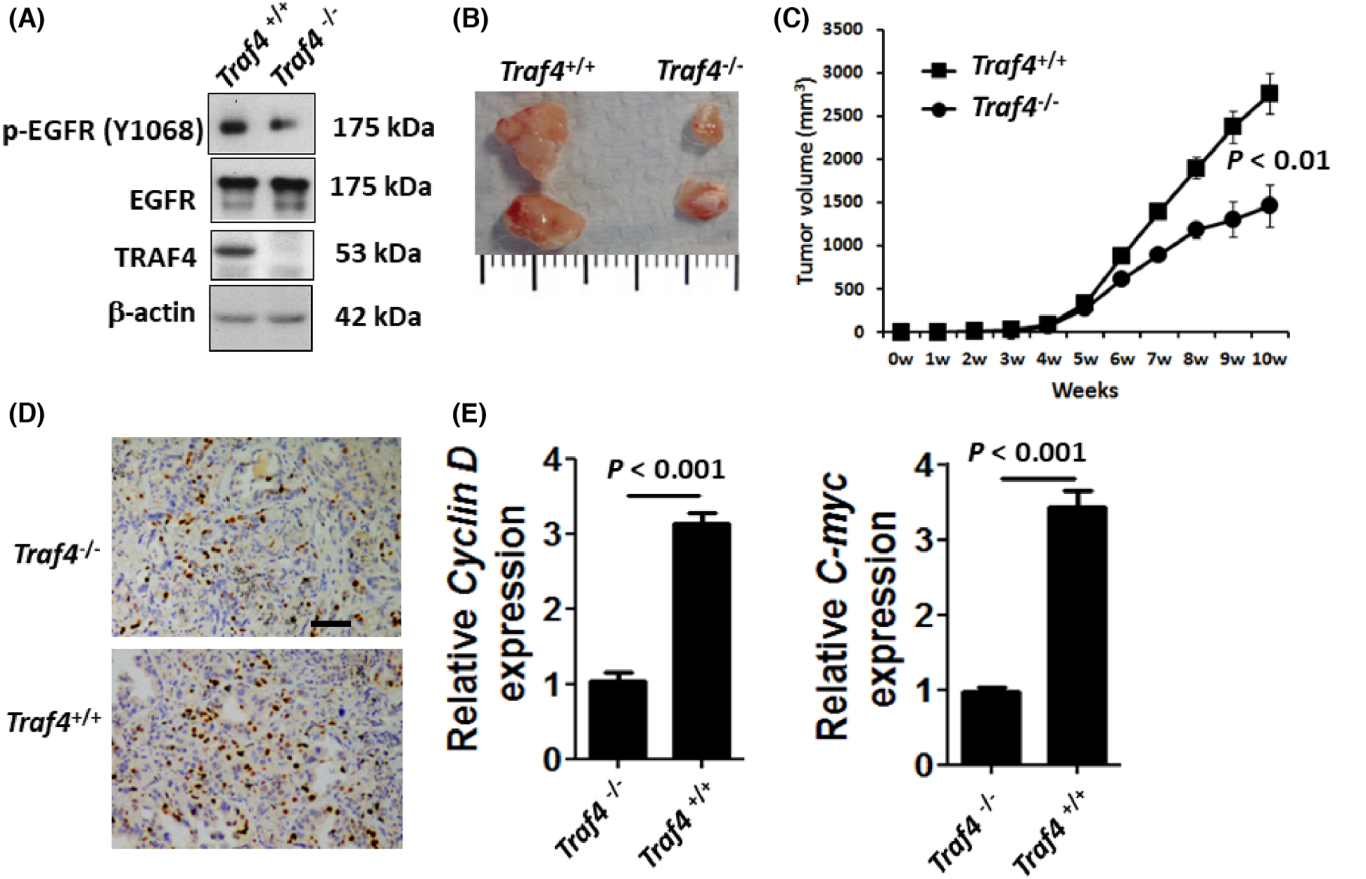
TRAF4 regulates tumor growth *in vivo*. (A) Pieces of tumors were homogenized, subjected to western blotting, and analyzed using antibodies as indicated. (B) Representative tumors analyzed in this assay are shown. (C) Primary tumor growth was measured upon orthotopic injection of TRAF4 KO(*Traf4* ^−/−^) cells or WT (*Traf4* ^+/+^) control cells. The experiment was terminated 10 weeks after injection (*n* = 5 per group per timepoint). Each timepoint shows the means ± standard errors of the means (SEM) of the results. Statistical significance was determined by Student's *t* test. (D) Representative Ki67 staining in exogenous tumor tissues. At 10 weeks after orthotopic implantation of A549 cells with or without TRAF4 KO, tumor tissues were done with immunohistochemical staining of Ki67. Scale bar, 50 μm. (E) Relative Cyclin D and C‐myc expression levels in TRAF KO and WT tumor tissues were evaluated by RT‐PCR. Statistical significance was determined by Student's *t* test.

To confirm the oncogenic role of TRAF4 in the expansion of lung cancer cells, we initiated *Traf4* ^−/−^ cell from the lung cancer cell line A549 via the Crisper/Cas9 lentivirus system and studied their growth in animal models. Single *Traf4* ^−/−^ and wildtype (WT) (*Traf4* ^+/+^) clones were symmetrically inoculated into the peritoneum of both sides of immune‐deficient NSG (NOD.Cg‐*Prkdc* ^*scid*^ *Il2rg* ^*tm1Wjl*^ */SzJ*) mice. Mice were monitored for tumor growth weekly and were sacrificed at 10 weeks after injection. Tumor tissues were subjected to western blotting to confirm TRAF4 protein levels. As expected, control tumors still expressed TRAF4, while those generated from *Traf4* ^−/−^ cells did not (Fig. 2A). The results showed that the growth rate of the *Traf4* ^−/−^ tumor cells was reduced by around 50% (Fig. 2B,C). By the growth rate, *Traf4* ^−/−^ tumor cells had a significantly decreased level of Ki67 (Fig. 2D) and reduced mRNA expression of *Cyclin D* and *C‐myc* (Fig. 2E). Considering the findings from clinical and experimental data, we conclude that TRAF4 plays a novel and essential role in facilitating lung cancer cell proliferation.

### EGFR is critical to TRAF4‐mediated proliferation of tumor cell

**Fig. 3:**
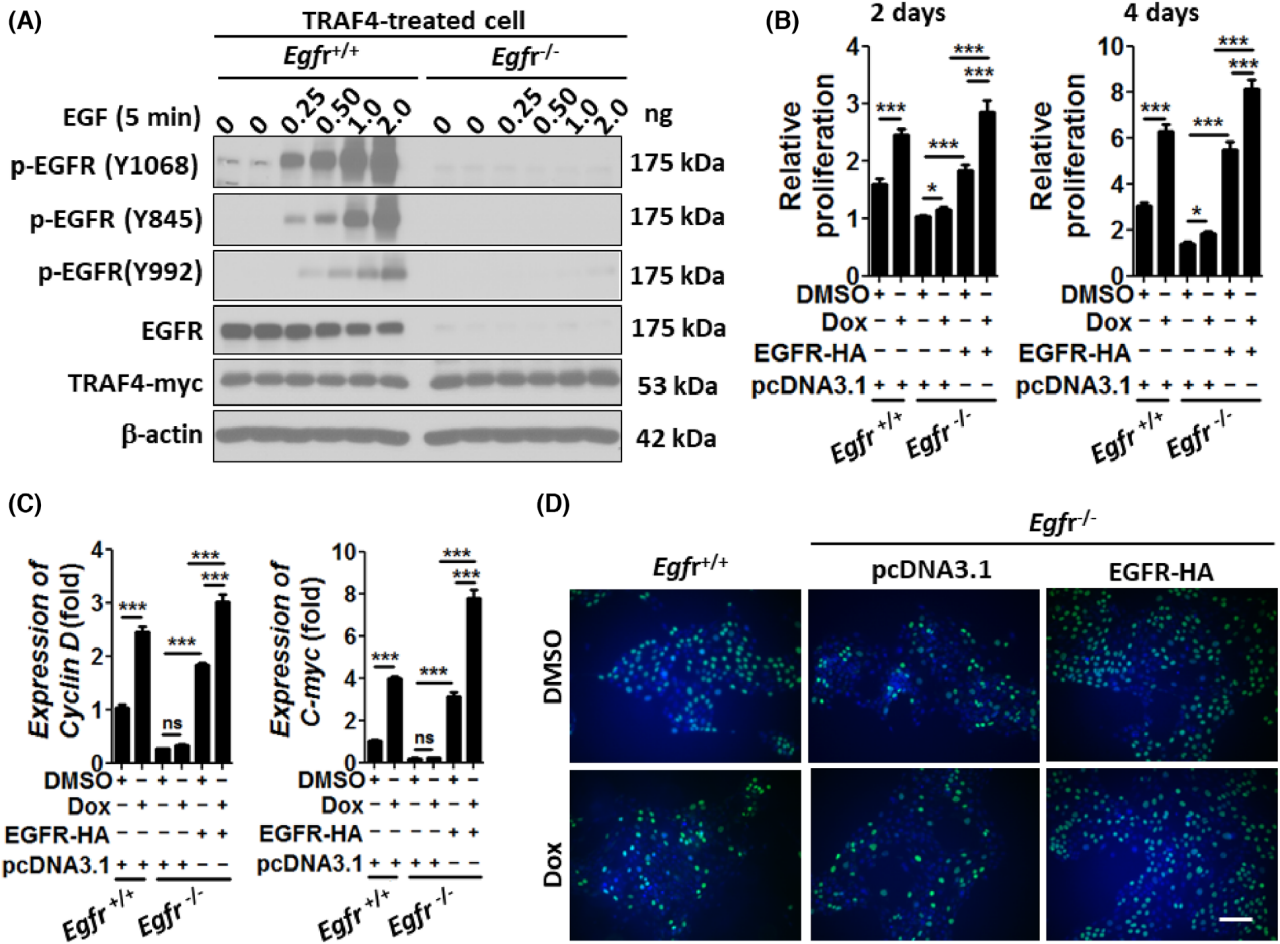
The TRAF4‐mediated cell proliferation is abolished in EGFR null cells. (A) *Egfr* ^−/−^ TRAF4‐treated A549 cells and *Egfr* ^+/+^ TRAF4 inducible A549 cells were treated with doxycycline to induce TRAF4 expression. After 24 h of treatment, serum‐starved cells were stimulated with EGF for 5 min. Cell lysates were subjected to western blot using antibodies as indicated. (B) EGFR‐HA expression plasmid or control plasmid transfected *Egfr* ^−/−^ TRAF4 inducible A549 cells, as well as *Egfr* ^+/+^ TRAF4 inducible A549 cells, were inoculated in 96‐well plates at a density of 500/well, in the presence of doxycycline or DMSO. The cells proliferation was monitored by a CCK8 assay. Statistical significance was determined by a one‐way ANOVA. The error bars represent SEM. *P < 0.05, ***P < 0.001. (C) Cyclin D and C‐myc mRNA was determined by RT‐PCR in the cell as described in panel B. Statistical significance was determined by a one‐way ANOVA. The error bars represent SEM. ***P < 0.001. (D) BrdU assay was performed in cells as described in panel C. Scale bar, 10 μm. The data are a representation of three independent experiments.

We previously demonstrated that TRAF4 activates EGFR by promoting its asymmetric dimerization [10]. To confirm the critical role of EGFR in TRAF4‐mediated proliferation of lung cancer cells, we deleted the *Egfr* gene in the TRAF4 inducible A549 cell line. Western blotting data demonstrated that this cell totally abolished the phosphorylation of EGFR upon EGF stimulation, even under the condition of TRAF4 overexpression (Fig. 3A). TRAF4 failed to induce *Egfr* ^−/−^ cell proliferation unless it receives the transgenic expression of exogenous EGFR (Fig. 3B). Additionally, depletion of EGFR decreased TRAF4‐mediated *Cyclin* D and *C‐myc* expression (Fig. 3C) and incorporation of BrdU (Fig. 3D). These results clearly described the role of EGFR in TRAF4‐mediated cancer c7ell proliferation.

### Enhanced phosphorylation of ERK5 in TRAF4‐overexpressing tumor cells

**Fig. 4:**
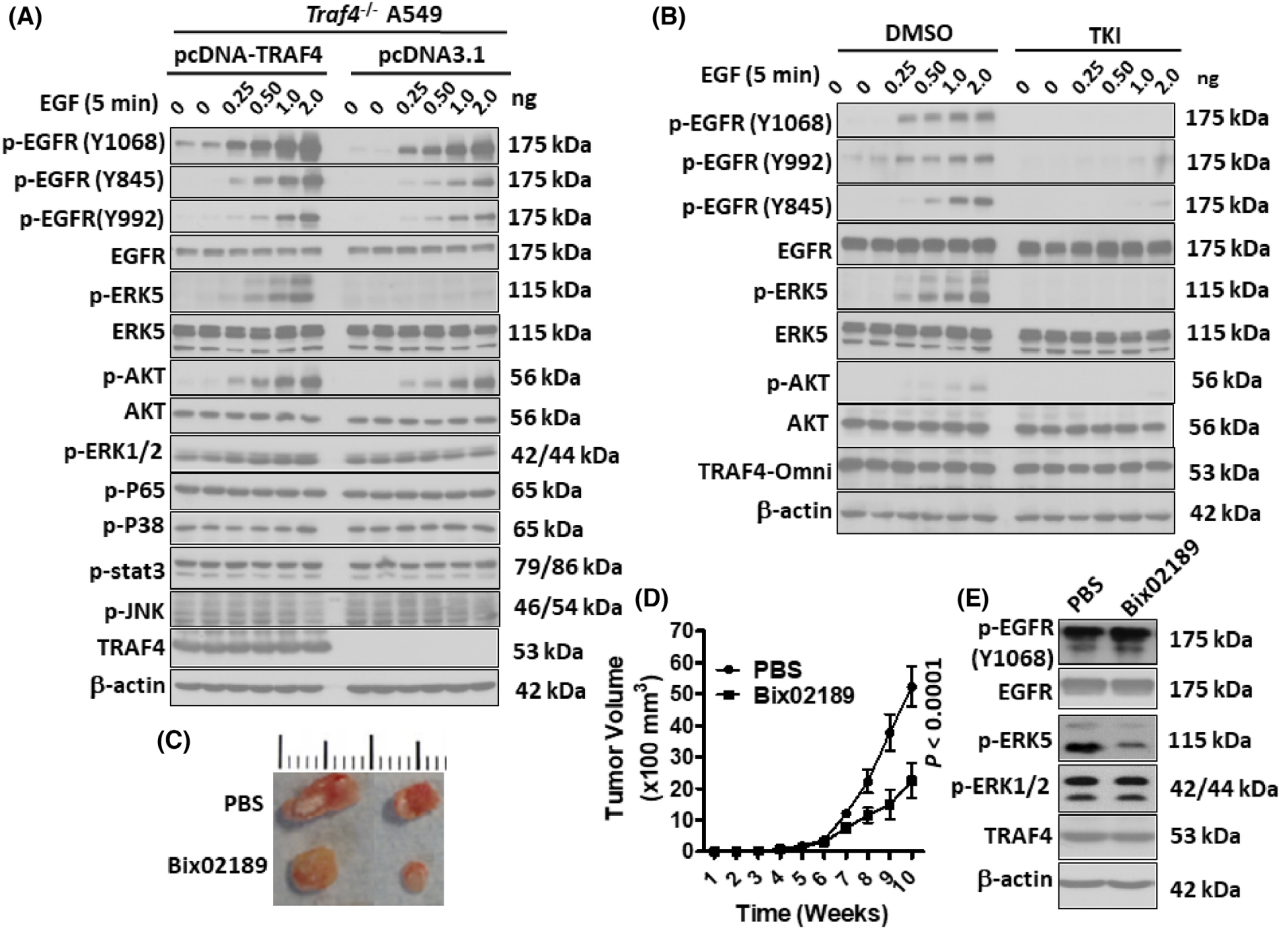
Overexpression of TRAF4‐enhanced phosphorylation of ERK5. (A) *Traf4* ^*−/−*^ A549 cells were transfected with TRAF4wt expression plasmid or control pcDNA3.1 plasmid. After 48 h, cells were stimulated with a low concentration of EGF (0.25, 0.5, 1.0, 2.0 ng·mL^−1^) for another 5 min. Cell lysates were subjected to western blot analysis using the indicated antibodies. (B) Serum‐starved TRAF4 inducible A549 cells were treated with the TK inhibitor (TKI) or DMSO for 24 h, and then stimulated with a low concentration of EGF (0.25, 0.5, 1.0, 2.0 ng·mL^−1^) for another 5 min. Cell lysates were subjected to western blot analysis using the indicated antibodies. (C) Primary tumor growth was measured upon orthotopic injection of A549 control cells. After cells injection, mice were treated with PBS or MEK5 inhibitor (Bix02189) every 2 days at a concentration of 5 μm. The experiment was terminated 10 weeks after injection (*n* = 5 per group per timepoint). The representative tumors are shown. (D) The tumor size was evaluated every week. Each timepoint shows the means ± standard errors of the means (SEM) of the results. Statistical significance was determined by Student's *t* test. (E) Pieces of tumors were homogenized, subjected to western blotting, and analyzed using antibodies as indicated.

To investigate the signaling mechanism of EGFR/TRAF4 in tumor cells, *Traf4* ^−/−^ cells were stimulated with EGF in the presence or absence of exogenous TRAF4. As shown in Fig. 4A, activation of ERK5 and, to a lesser extent, Akt (but not ERK1/2, P38, P65, stat3, and JNK) was enhanced by TRAF4 expression. We also demonstrated that TRAF4‐mediated activation of ERK5 and Akt was critically dependent on EGFR activation, since the tyrosine kinase inhibitor (TKI) of EGFR (Gefitinib) and inactive mutant (EGFR‐D813N) failed to phosphorylate downstream ERK5 and AKT signaling (Fig. 4B and Fig. S2A). Additionally, ERK5 phosphorylation was independent of AKT activation, since no significant alteration was found in EGFT/TRAF4‐mediated phosphorylation of ERK5 in the presence of an AKT inhibitor (sc‐394,003) (Fig. S2B). Although ERK5 signaling is one of the less‐studied MAPK cascades, there has been increasing evidence showing that ERK5 signaling is involved in cell proliferation and tumor development and progression. Thus, we explored the role of EGFR/TRAF4‐mediated ERK5 activation in the proliferation of lung cancer cells. Genetic inhibition of ERK5 activation by a specific siRNA of ERK5 markedly reduced the growth of TRAF4‐overexpression A549 cells (Fig. S3A), as well as the expression levels of *Cyclin* D and *C‐myc* mRNA (Fig. S3B). To support this finding, we treated TRAF4‐sufficient A549 xenograft models with an MEK5 inhibitor (Bix02189), which blocks ERK5 activation [20], and monitored tumor growth. Suppression of ERK5 drastically reduced the tumor size (Fig. 4C,D). Western blot analysis of tumors dissected at the end of the experiments confirmed the reduction of phosphorylated ERK5 in the tissue from Bix02189‐treated mice (Fig. 4E).

### TRAF4 bridges MEKK3 to EGFR through the TRAF domain

**Fig. 5:**
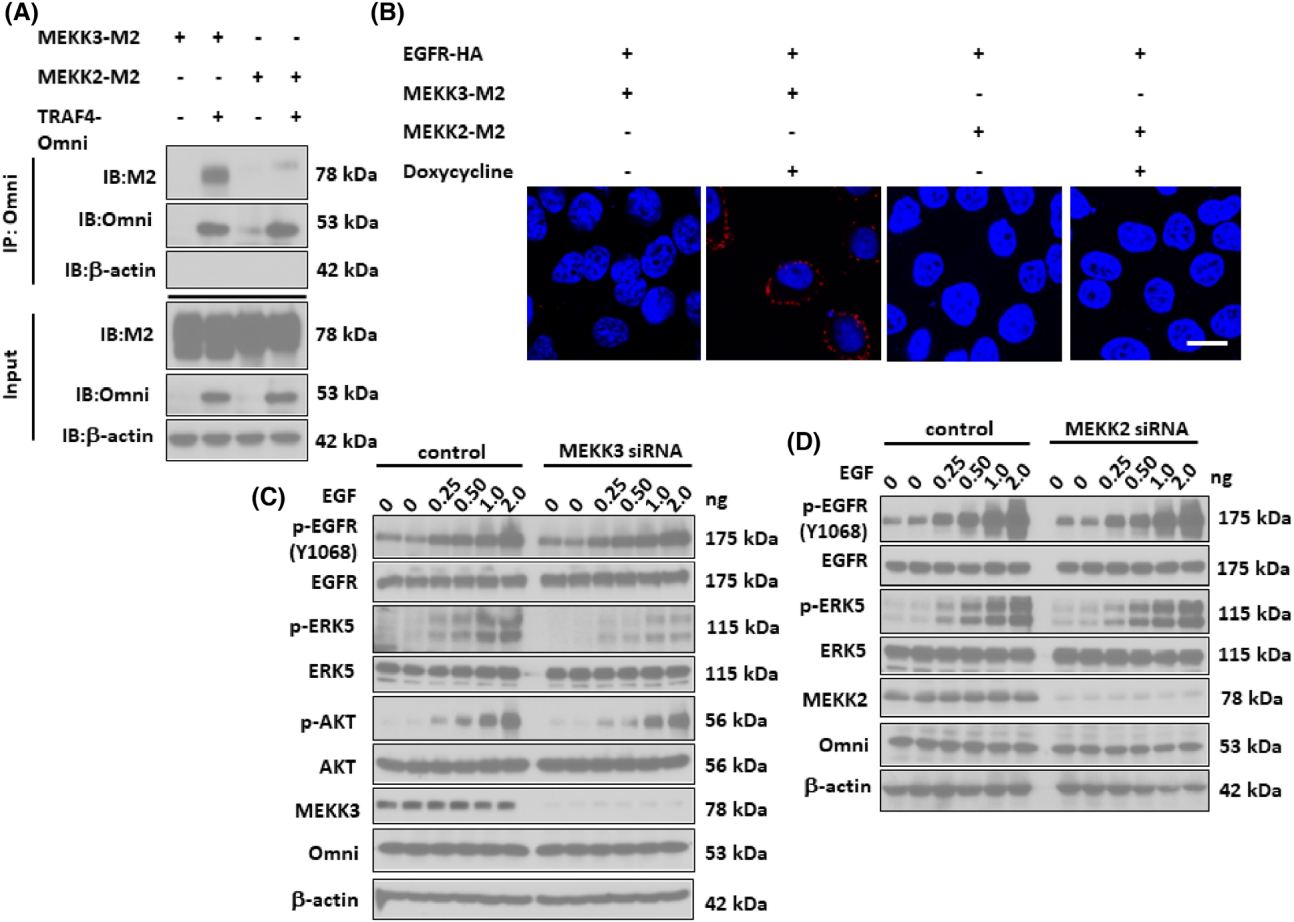
TRAF4 bridges MEKK3 to EGFR. (A) HEK‐293T cells were cotransfected with the pcDNA‐TRAF4‐Omni plasmid and pcDNA‐MEKK3‐M2, pcDNA‐MEKK2‐M2, or control pcDNA3.1 plasmid as indicated. Cell lysates were then immunoprecipitated with anti‐Omni antibody, followed by western blot analysis. (B) pcDNA‐EGFR‐HA plasmid was cotransfected with pcDNA‐MEKK3‐M2 or pcDNA‐MEKK2‐M2 plasmid into A549 cells in the presence of doxycycline or DMSO and fixed for detecting the interaction between M2 and HA through the Duolink experiment according to the instructions of the manufacturer. Scale bar, 5 μm. (C) TRAF4‐treated A549 cells were transfected with siRNA target to MEKK3 or control siRNA. Cells were then treated with doxycycline followed by EGF stimulation. Cell lysates were collected for western blot experiments using indicated antibodies. (D) TRAF4 inducible A549 cells were transfected with siRNA target to MEKK2 or control siRNA. Cells were then treated with doxycycline followed by EGF stimulation. Cell lysates were collected for western blot experiments using indicated antibodies.

ERK5, also known as big MAPK, is the least‐studied MAPKs [21]. It is activated by its upstream MEK5, which in turn is activated by MEKK2 or MEKK3. We found that TRAF4 immunoprecipitated with MEKK3, rather than MEKK2, upon overexpression (Fig. 5A). Since we previously found that EGFR interacts with TRAF4 [10], we supposed that TRAF4 bridges EGFR to MEKK3 and promotes activation of MEKK3. Through Duolink proximity ligation assay, we confirmed that EGFR recruited MEKK3 in a TRAF4‐dependent manner (Fig. 5B). Genetic suppression of MEKK3 by specific siRNA dramatically inhibited ERK5 activation (Fig. 5C) and cell proliferation (data not shown). However, activation of ERK5 triggered by EGFR/TRAF4 signaling was almost intact in the absence of MEKK2 (Fig. 5D). In sum, these data suggest a critical role of MEKK3 in mediating EGFR/TRAF4 signaling to the phosphorylation of ERK5.

### Structural mechanism of TRAF4 in bridging EGFR to TRAF4

**Fig. 6:**
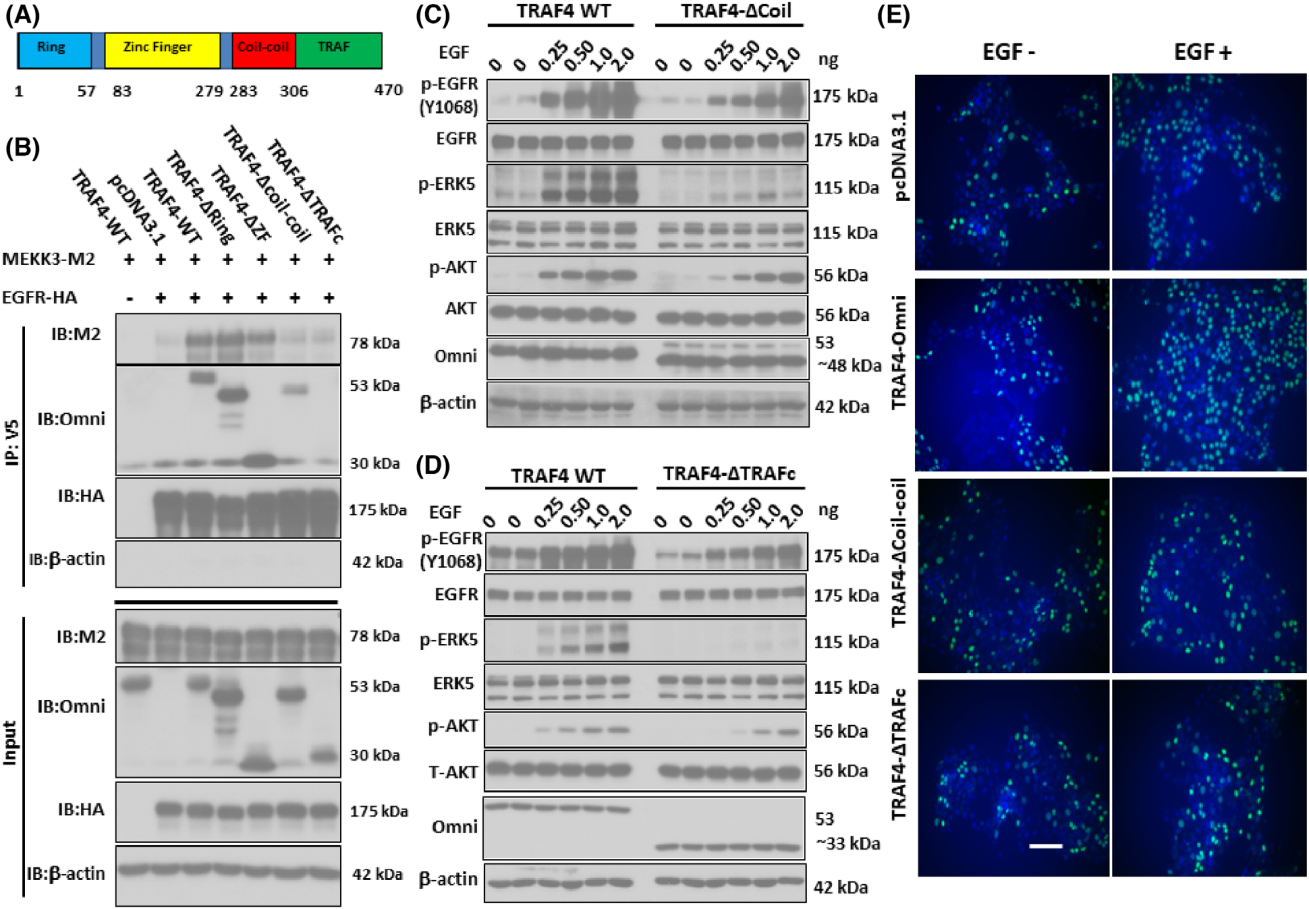
TRAF4 bridges MEKK3 to EGFR through the Coil‐coil domain and TRAFc domain. (A) Domain structure of TRAF4. (B) TRAF4 KO cells were transfected with plasmids encoding EGFR‐HA and MEKK3‐M2 in the presence or absence of different domain‐deleted TRAF4‐Omni truncates. After 48 h of transfection, cell lysates were subjected to immunoprecipitation with anti‐HA antibody, followed by western blot analysis using different antibodies, as indicated. (C) TRAF4 KO A549 cells were transfected with plasmids encoding whole‐length TRAF4‐Omni or TRAF4 deltaCoil‐coil‐Omni. After 48 h of transfection, the serum‐starved cell was stimulated by EGF for 5 min. Cell lysates were subjected to western blot analysis using indicated antibodies. (D) TRAF4 KO A549 cells were transfected with plasmids encoding whole‐length TRAF4‐Omni or TRAF4 deltaTRAFc‐Omni. After 48 h of transfection, serum‐starved cells were stimulated by EGF for 5 min. Cell lysates were subjected to western blot analysis using indicated antibodies. (E) TRAF4 KO A549 cells were transfected with plasmids encoding whole‐length TRAF4‐Omni, TRAF4‐deltaTRAFc‐Omni, or TRAF4 deltaCoil‐coil‐Omni. BrdU assay was performed in cells as described in panel C. Scale bar, 10 μm. The data are a representation of three independent experiments.

The TRAF4 protein consists of four structural domains, from N‐terminal to C‐terminal, including a Ring domain, a Zinc Finger (ZF) domain, a coil‐coil domain, and a TRAF domain [22]. We initiated TRAF4‐expressing vectors that were deleted; the specific sequence is labeled in Fig. 6A. We firstly demonstrated that the TRAFc domain is necessary for TRAF4 to interact with MEKK3 (Fig. S4A). Although deletion of the Ring domain and the ZF domain had no impact on the EGFR‐MEKK3 interaction, removal of the TRAF domain and the Coil‐coil domain abolished the EGFR‐MEKK3 interaction (Fig. 6B). Moreover, TRAF4 proteins without either the Coil‐coil domain or the TRAFc domain failed to enhance phosphorylation of both EGFR and ERK5 upon stimulation (Fig. 6C,D). Besides, TRAF4‐mediated cancer cell proliferation (Fig. S4B), expression of *Cyclin D* and *C‐myc* (Fig. S4C), as well as the incorporation of BrdU (Fig. 6E), were accordingly abolished when the TRAFc domain or the Coil‐coil domain was deleted. However, neither the Ring domain nor the ZF domain truncations affected the activation of EGFR and ERK5 (Fig. S5A,B). In sum, TRAF4 bridges EGFR and MEKK3 and promotes phosphorylation of ERK5 through the TRAF domain.

### TRAF4‐ERK5 is hyperactivated in human NSCLC

**Fig. 7:**
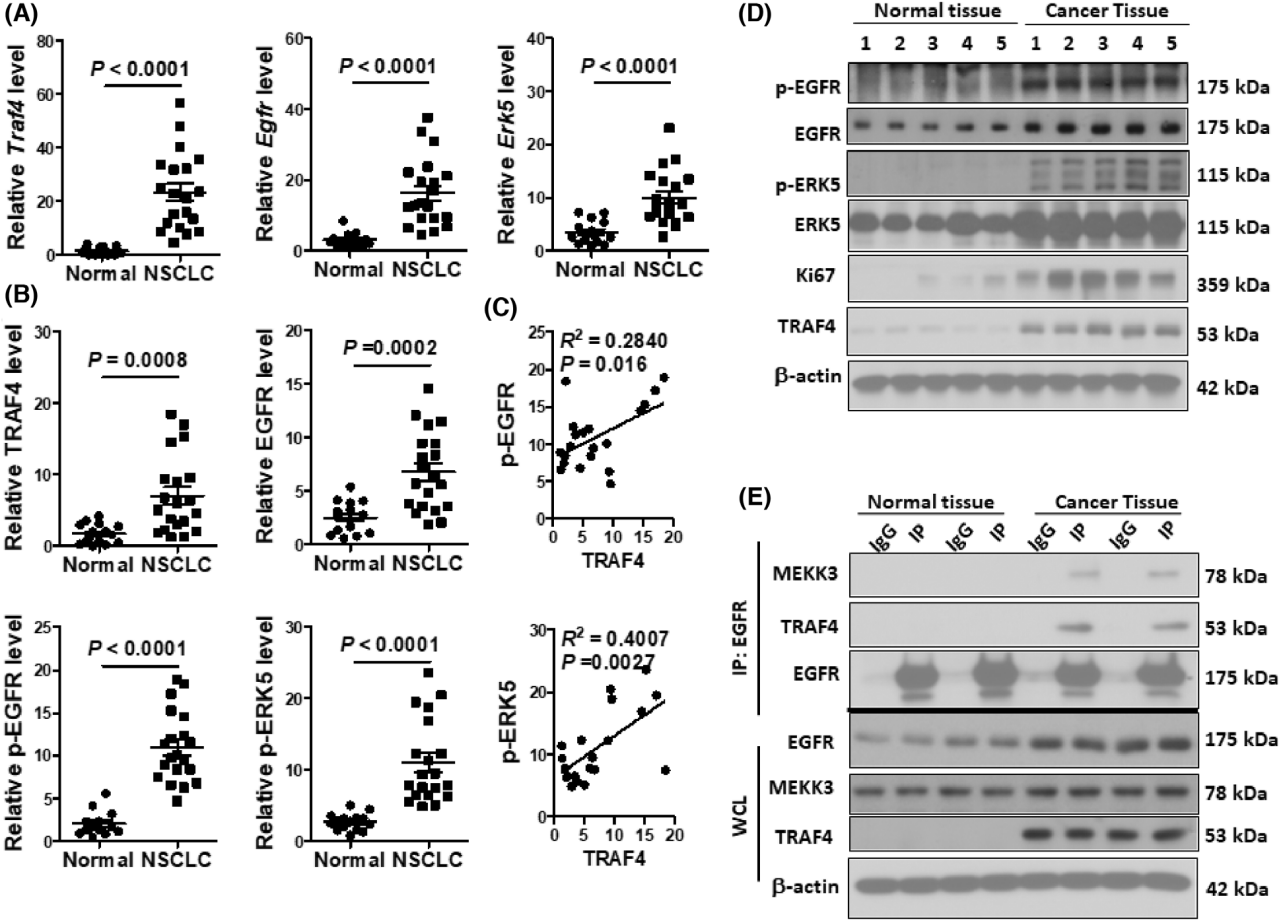
The EGFR‐TRAF4–ERK5 is a dominant pathway in human NSCLC. (A) RT‐PCR analysis from FFPE sections of human normal (*n* = 15) or NSCLC samples (*n* = 20). Gene expression is graphed as a relative fold in NSCLC over normal lung. Each dot represents an independent sample. (B) Densitometric quantification of the indicated proteins in lysates of human normal lung tissue (*n* = 15) or NSCLC (*n* = 20) as analyzed by western blot. (C) Linear regression of the indicated protein levels in NSCLC as detected by western blot. (D) Representative results of western blot analysis of normal human lung tissue or NSCLC samples. Each lane represents samples from an individual patient. (E) Freshly collected normal human lung tissue or NSCLC samples were lysed and subjected to immunoprecipitation with anti‐EGFR antibody, followed by western blot analysis. Each pair of lanes (IgG, IP) represents a sample from an individual patient. All the data are representative of three experiments.

TRAF4 has long been found to be overexpressed in various carcinomas [6], whereas EGFR has been shown to be associated with NSCLC [1, 8, 9, 23]. *Egfr* and *traf4* were indeed overexpressed at both the RNA and protein levels in human NSCLC samples compared to normal lung tissue (Fig. 7A,B). More important, the overexpression of TRAF4 was strongly correlated with the activation of p‐ERK5 and the level of phosphorylated EGFR (Fig. 7C,D). Additionally, coimmunoprecipitation experiments showed that the EGFR‐TRAF4‐MEKK3 complex was readily detectable in NSCLC lysates but not in normal lung tissue, indicating the activation of the EGFR‐TRAF4‐MEKK3/ERK5 pathway (Fig. 7E). Notably, the level of phosphorylated EGFR was correlated with the TRAF4 expression in NSCLC (Fig. 7C), implicating the EGFR signaling in NSCLC with the overexpression of TRAF4. Collectively, our data imply that the emergence of TRAF4 allows EGFR activation to engage the MEKK3‐ERK5 axis, promoting tumor formation through the induction of genes critical for cell proliferation.

## Discussion

TRAF4 was initially identified in human breast cancer in 1995 [4]. In the past few years, multiple studies revealed that TRAF4 is engaged in several signaling pathways and has essential biological functions in the generation and development of carcinoma. For instance, TRAF4 is involved in cell transformation through mediating ubiquitination and activating Akt [7]. TRAF4 plays a vital role in activating the p70s6k/S6 signaling pathway and promoting cell proliferation [24]. TRAF was also found to regulate the Wnt/catenin pathway in colon cancer [25] and oral squamous cell carcinoma [26]. Recently, a study revealed that TRAF4 interacts with EGFR to promote asymmetric dimerization of EGFR [10]. In this study, we first set up the relationship between robust proliferation, as indicated by positive staining of Ki67, and TRAF4 expression levels in clinical NSCLC tissue cells. Moreover, by performing a retrospective survey the TRAF4 expression was found to be associated with PFS in stage IIIa NSCLC patients. These data suggest that the increased level of TRAF4, which was commonly observed in lung cancer, has functional consequences. In subsequent experiments, both *in vivo* and *ex vivo* data demonstrated that TRAF4 strongly promoted the expansion of cancer cells, clearly indicating an oncogenic role of TRAF4 on lung cancer.

EGFR‐mediated signaling transduction is always deregulated in lung cancer. The uncontrollable activation of EGFR notably accelerates malignant progression and induces drug resistance. In this study, we also confirmed TRAF4 as an accelerator of EGFR activation and its critical role in tumor genesis of NSCLC, as per the published data [10]. Downstream kinases of EGFR, including (but not limited to) the mitogen‐activated protein kinases (MAPKs) and PI3K‐Akt, are always hyperactive because of abnormal auto‐phosphorylation of EGFR in tumor tissue. MAPKs are classified into four main groups, including ERK1/2, JNK, p38, and ERK5 [27]. To further characterize the EGFR/TRAF4 signaling pathway, the overactivation of downstream kinase was detected. To our surprise, we found that phosphorylation of ERK5, but not other MAPKs, was tightly associated with TRAF4 expression, as well as active EGFR. We also found that the phosphorylation of Akt was involved in TRAF4‐mediated EGFR activation, whereas it was not required for ERK5 phosphorylation. Previous studies showed that the TRAF4‐mediated ubiquitination and the activation of Akt were involved in the cellular glycolysis of cancer cells [7]. Here we revealed that the phosphorylation of ERK5 was closely associated with cell proliferation. Thus, these data suggest that abnormal EGFR/TRAF4 signaling cascades have multiple effects on promoting tumor progress.

ERK5, as well as other MAPKs, are the terminal kinase of a three‐kinase activation cascade system, in which MAPKs was phosphorylated and consequently activated by a MAPK kinase (MAP2K), and then phosphorylated and activated by a MAPK kinase kinase (MAP3K). While the ERK1/2, p38, and JNK MAPK are all responsive to activation signals originating from several different MAP3K, ERK5 activity is shown to be predominantly under the control of EKK2 and MEKK3 members of the MAP3K class [27]. Both MEKK2 and MEKK3 phosphorylate and activate MAP2K protein MEK5, and then MEK5 phosphorylates and activates ERK5 [27]. Here we showed that TRAF4 immunoprecipitated MEKK3 rather than MEKK2, suggesting that MEKK3 plays a crucial role in EGFR/TRAF4‐mediated phosphorylation of ERK5. Subsequent experiments confirmed TRFAF4 as a bridge connecting EGFR and MEKK3. Our finding in the Duolink proximity ligation assay revealed a new insight into the regulation of MEKK3 signaling. In the presence of TRAF4, the predominantly cytoplasmic MEKK3 is translocated to membrane‐proximal areas, which strongly suggests that the formation of the EGFR/TRAF4 signaling complex can recruit MEKK3.

After confirming the role of TRAF4 in the formation of the EGFR‐TRAF4‐MEKK3 signaling complex, we investigated how TRAF4 facilitates the formation of such a complex. We first demonstrated that the TRAFc domain is the binding domain of MEKK3 (Fig. S4A), and that this domain is necessary for TRAF4‐mediated phosphorylation of EGFR and ERK5 (Fig. 6C). In addition to the TRAFc domain, the Coil‐coil domain also shows its importance (Fig. 6B). Previous crystallographic and functional studies have demonstrated that TRAF4, like other TRAF family members, assemble as a trimer in both solution and crystals. The trimer is the smallest functional unit of the TRAF family [28]. Structural analysis shows that the C‐terminal (including the Coil‐coil domain and TRAFc domain) of the TRAF4 trimer is composed of a “stalk” region (the Coil‐coil domain) and a “cap” region (the TRAFc domain). The Coil‐coil domain acts as a hinge to combine the TRAF4 monomers [22, 28]. Since the TRAFc domain is either an MEKK3‐ (Fig. S5) or EGFR‐binding domain [10], we speculated that TRAF4 could act as a multivalent adaptor for clustering of EGFR and MEKK3. As shown in Fig. 6, deletion of either the TRAFc domain or the Coil‐coil domain failed to mediate the interaction between EGFR and MEKK3. These data provide confirmative evidence that the trimerization of TRAF4 is essential for TRAF4‐mediated ERK5 activation, and the role of TRAF4 is to bridge the EGFR and MEKK3 proteins.

Notably, TRAF4 is overexpressed in a wide range of human malignancies, with 70% of tumors overexpressing TRAF4 without altering gene copy numbers [17]. We indeed found that *Traf4* was overexpressed in human NSCLC, in which we detected the activation of EGFR, the EGFR‐TRAF4‐MEKK3 complex, and strong ERK5 phosphorylation, indicating that the signaling axis is hyperactivated in human NSCLC. ERK5 and EGFR also showed increased protein levels in NSCLC, and we hypothesized that the EGFR‐TRAF4‐MEKK3‐ERK5 signal axis might have positive feedback on the expression of ERK5 and EGFR. This may require further investigation in the future. However, according to our data, we propose that the overexpression of TRAF4 could be an essential feature of tumor cells, where an elevated TRAF4 expression induces activation of the EGFR‐TRAF4‐MEKK3‐ERK5 axis as the dominant pathway to promote tumorigenesis.

In this study our data strongly suggest that TRAF4 has a putative oncogene function in lung cancer. By reducing ERK5 phosphorylation, RAF4 deletion attenuates NSCLC cell proliferation. TRAF4 acts as an adaptor for EGFR to interact with MEKK3, a kinase that activates ERK5. The EGFR‐TRAF4‐MEKK3‐ERK5 axis is widely present in NSCLC, suggesting its critical role in tumorigenesis. Therefore, the results of this study not only set up a clear clinical association of TRAF4, but also elucidate an abnormal signaling pathway for tumor cell proliferation. It will be a potential target for therapeutic intervention of NSCLC.

## Conflict of interest

The authors declare no conflict of interest.

## Author contributions

GC and XN conceptualized and supervised the study. GC, SH, and DD designed the experiments. SH, JL, and BW performed the experiments and analyzed the data. SH and DD wrote the article. GC and XN revised the article.

## Supporting information


**Fig. S1.** The OS was analyzed between NSCLC patients with higher TRAF4 expression and those with lower expression level. Using 30% (left) or 40% (right) as the cutoff value of the TRAF4 level, the difference in overall survival (OS) between the two groups did not reach significance. The data analysis was performed with Prism 5.0.

**Fig. S2.** TRAF4‐mediated phosphorylation of ERK5 is dependent on activation of EGFR, but not Akt. (A) *Egfr* ^‐/‐^ A549 cells were transfected with pcDNA‐EGFRwt‐HA plasmid or pcDNA‐EGFR D813N‐HA for 48 h, and then stimulated with different concentrations of EGF (0.25, 0.5, 1.0, 2.0 ng/mL) for another 5 min. (B) TRAF4 over‐expressive A549 cells were treated with DMSO or Akt inhibitor (sc‐394003, 5 μM), followed by stimulation with EGF (0.25, 0.5, 1.0, 2.0 ng/mL) for another 5 min. Cell lysates were subject to Western blot analysis using indicated antibodies.

**Fig. S3.** Inhibition of ERK5 abolished cell proliferation. (A) TRAF4 overexpressed A549 cells were transfected with siRNA target to ERK5 or control siRNA. These cells were inoculated in 96 well plates at a density of 500/well in the presence or absence of EGF (1ng/mL). The cells proliferation was monitored by a CCK8 assay. Statistical significance was determined by a one‐way ANOVA. The error bars represented SEM. *** *P* < 0.001. (B) The relative expression level of Cyclin D and C‐myc mRNA was analyzed in cells as described in panel A. Statistical significance was determined by a one‐way ANOVA. The error bars represented SEM. * *P* < 0.05, ** *P* < 0.01, *** *P* < 0.001. The data is a representation of three independent experiments.

**Fig. S4.** TRAF4 interacts with MEKK3 through the TRAFc domain. (A) TRAF4 KO cells were transfected with plasmids encoding MEKK3‐M2, as well as different domain‐deleted TRAF4‐Omni truncates. Cells were collected and lysed after 48 h of transfection for subjecting to immunoprecipitation with anti‐Omni antibody, followed by Western blot analysis. All the data are representative of three experiments. (B) TRAF4 KO A549 cells were transfected with plasmids encoding whole length TRAF4‐Omni, TRAF4 deltaTRAFc‐Omni or TRAF4 deltaCoil‐coil‐Omni. These cells were inoculated in 96 well plates at a density of 500/well in the presence or absence of EGF (1ng/mL). The cells proliferation was monitored by a CCK8 assay. Statistical significance was determined by a one‐way ANOVA. The error bars represented SEM. *** *P* < 0.001. (C) The relative expression level of Cyclin D and C‐myc mRNA was analyzed in cells as described in panel B. Statistical significance was determined by a one‐way ANOVA. The error bars represented SEM. * *P* < 0.05, ** *P* < 0.01, *** *P* < 0.001. The data is a representation of three independent experiments.

**Fig. S5.** The role of Ring and ZF domain on TRAF4‐mediated EGFR/ERK5 activation. (A) TRAF4 KO A549 cells were transfected with plasmids encoding whole length TRAF4‐Omni or TRAF4 deltaRing‐Omni. (B) TRAF4 KO A549 cells were transfected with plasmids encoding whole length TRAF4‐Omni or TRAF4 deltaZF‐Omni. After 48 h of transfection, serum‐starved cells were stimulated by EGF for 5 min. Cell lysates were subjected to Western blot analysis using indicated antibodies. The data shown represent 3 or 4 independent experiments.

## Data Availability

The data that support the findings of this study are available from the corresponding author (caigangsmmu@hotmail.com) upon reasonable request.
