# Supplementary figures and images for: Overexpression of TRAF4 promotes lung cancer growth and EGFR‐dependent phosphorylation of ERK5
Source: FEBS Open Bio. 2022 Aug 17;12(10):1747–60. doi: 10.1002/2211-5463.13458 (PMC9527583; doi:10.1002/2211-5463.13458)

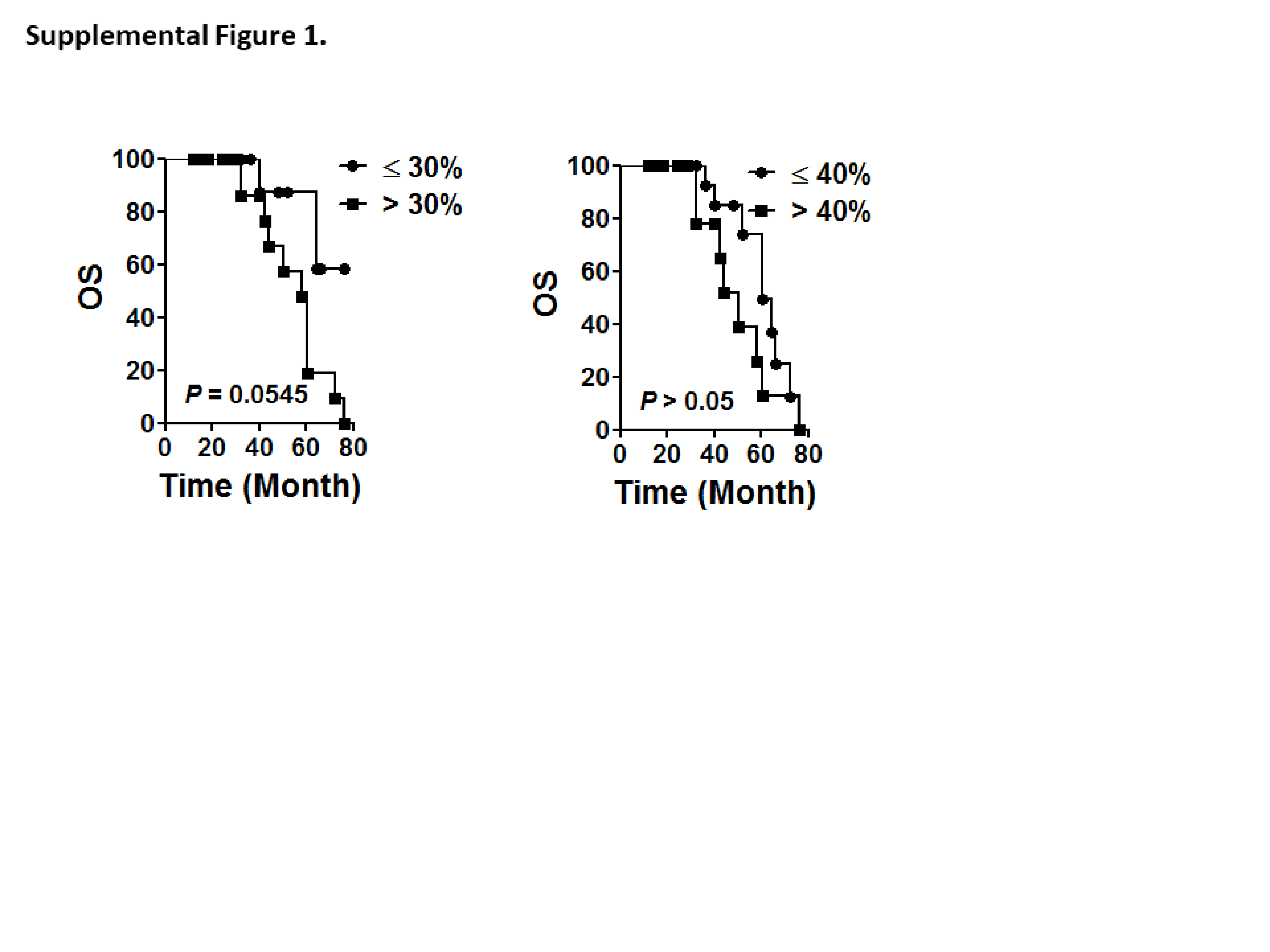

Supplement: Supplementary file 1 — Fig. S1. The OS was analyzed between NSCLC patients with higher TRAF4 expression and those with lower expression level. Using 30% (left) or 40% (right) as the cutoff value of the TRAF4 level, the difference in overall survival (OS) between the two groups did not reach significance. The data analysis was performed with Prism 5.0. [file FEB4-12-1747-s003.tif]

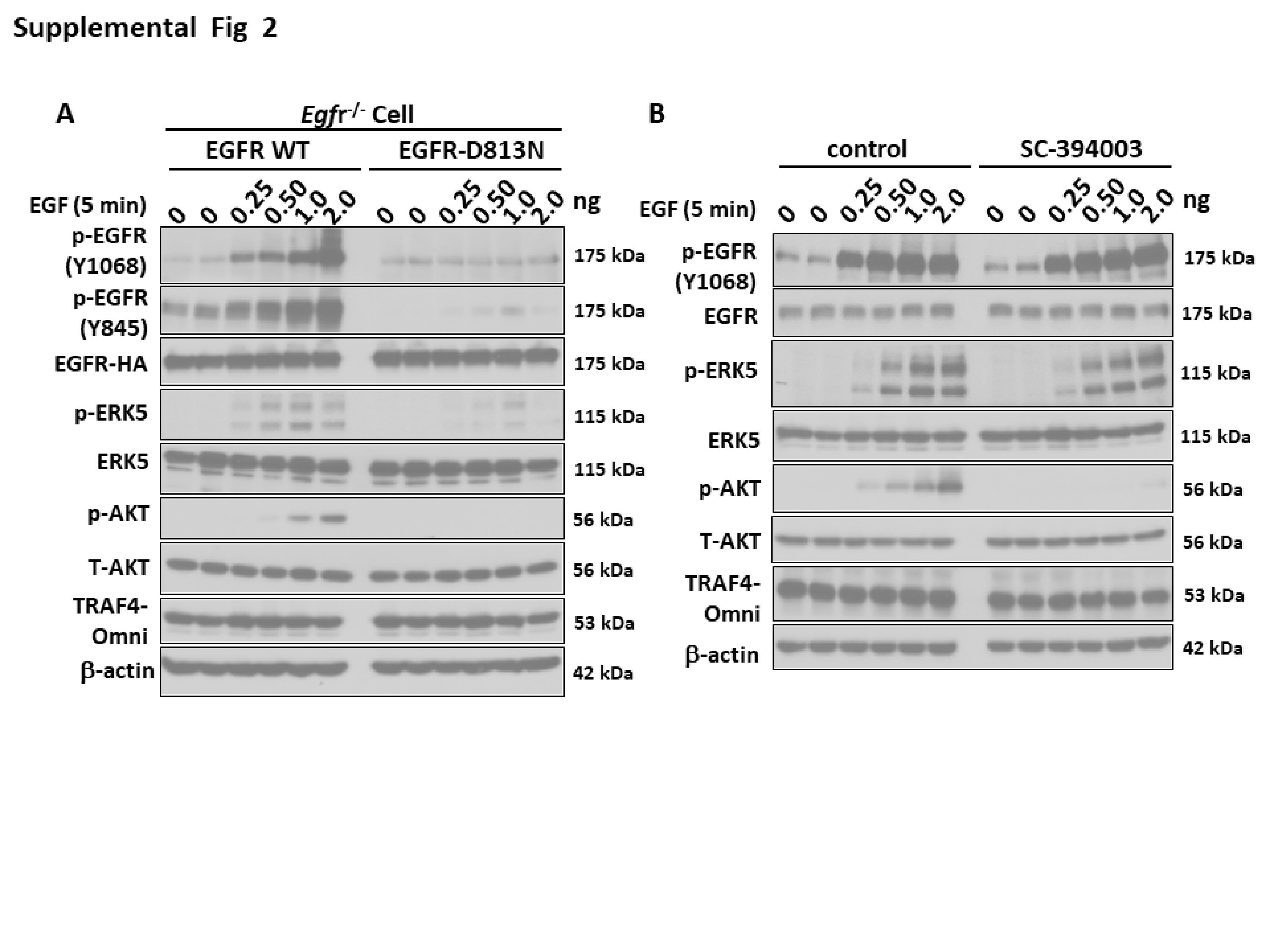

Supplement: Supplementary file 2 — Fig. S2. TRAF4‐mediated phosphorylation of ERK5 is dependent on activation of EGFR, but not Akt. (A) Egfr ‐/‐ A549 cells were transfected with pcDNA‐EGFRwt‐HA plasmid or pcDNA‐EGFR D813N‐HA for 48 h, and then stimulated with different concentrations of EGF (0.25, 0.5, 1.0, 2.0 ng/mL) for another 5 min. (B) TRAF4 over‐expressive A549 cells were treated with DMSO or Akt inhibitor (sc‐394003, 5 μM), followed by stimulation with EGF (0.25, 0.5, 1.0, 2.0 ng/mL) for another 5 min. Cell lysates were subject to Western blot analysis using indicated antibodies. [file FEB4-12-1747-s002.tif]

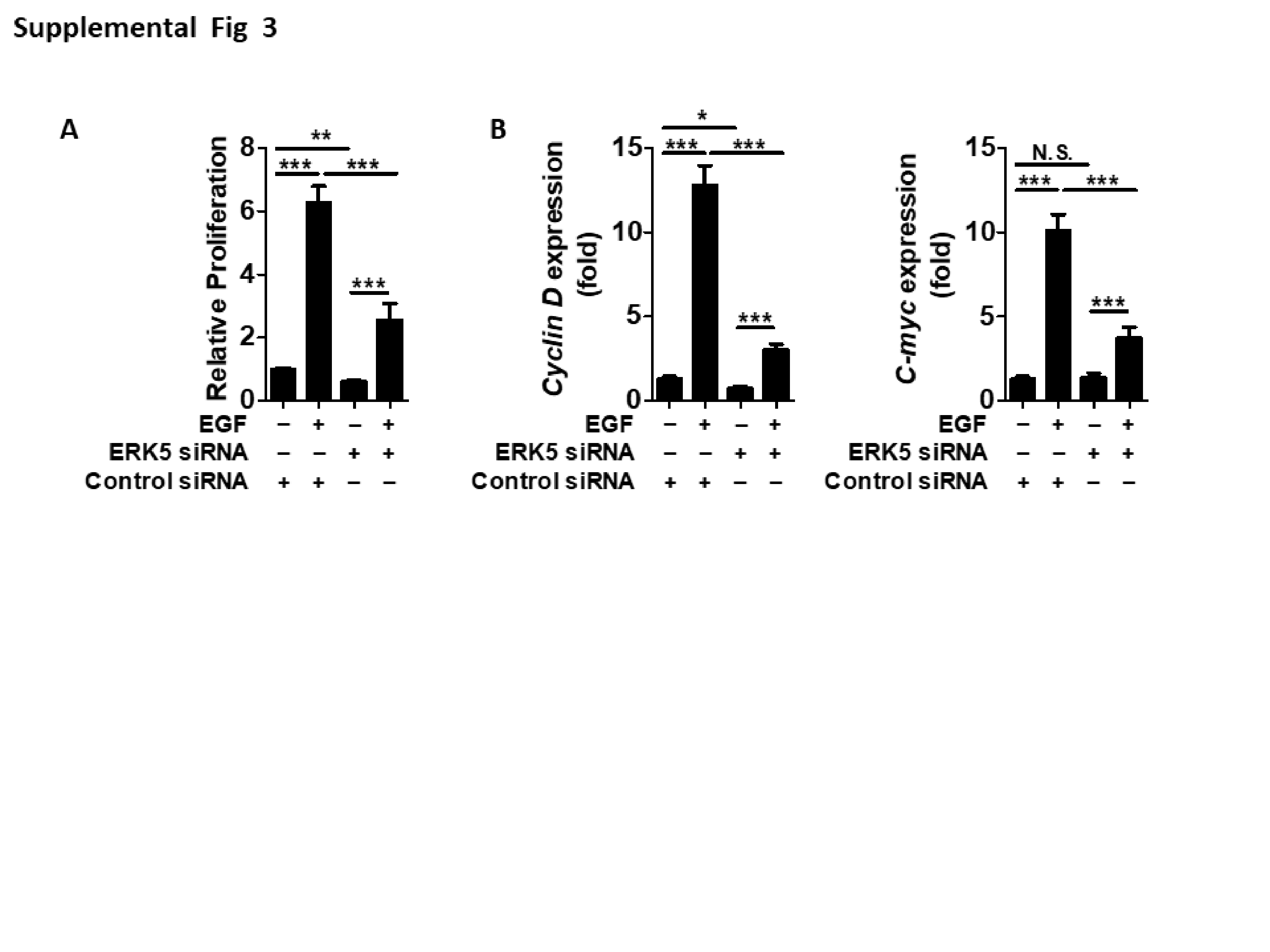

Supplement: Supplementary file 3 — Fig. S3. Inhibition of ERK5 abolished cell proliferation. (A) TRAF4 overexpressed A549 cells were transfected with siRNA target to ERK5 or control siRNA. These cells were inoculated in 96 well plates at a density of 500/well in the presence or absence of EGF (1ng/mL). The cells proliferation was monitored by a CCK8 assay. Statistical significance was determined by a one‐way ANOVA. The error bars represented SEM. *** P < 0.001. (B) The relative expression level of Cyclin D and C‐myc mRNA was analyzed in cells as described in panel A. Statistical significance was determined by a one‐way ANOVA. The error bars represented SEM. * P < 0.05, ** P < 0.01, *** P < 0.001. The data is a representation of three independent experiments. [file FEB4-12-1747-s005.tif]

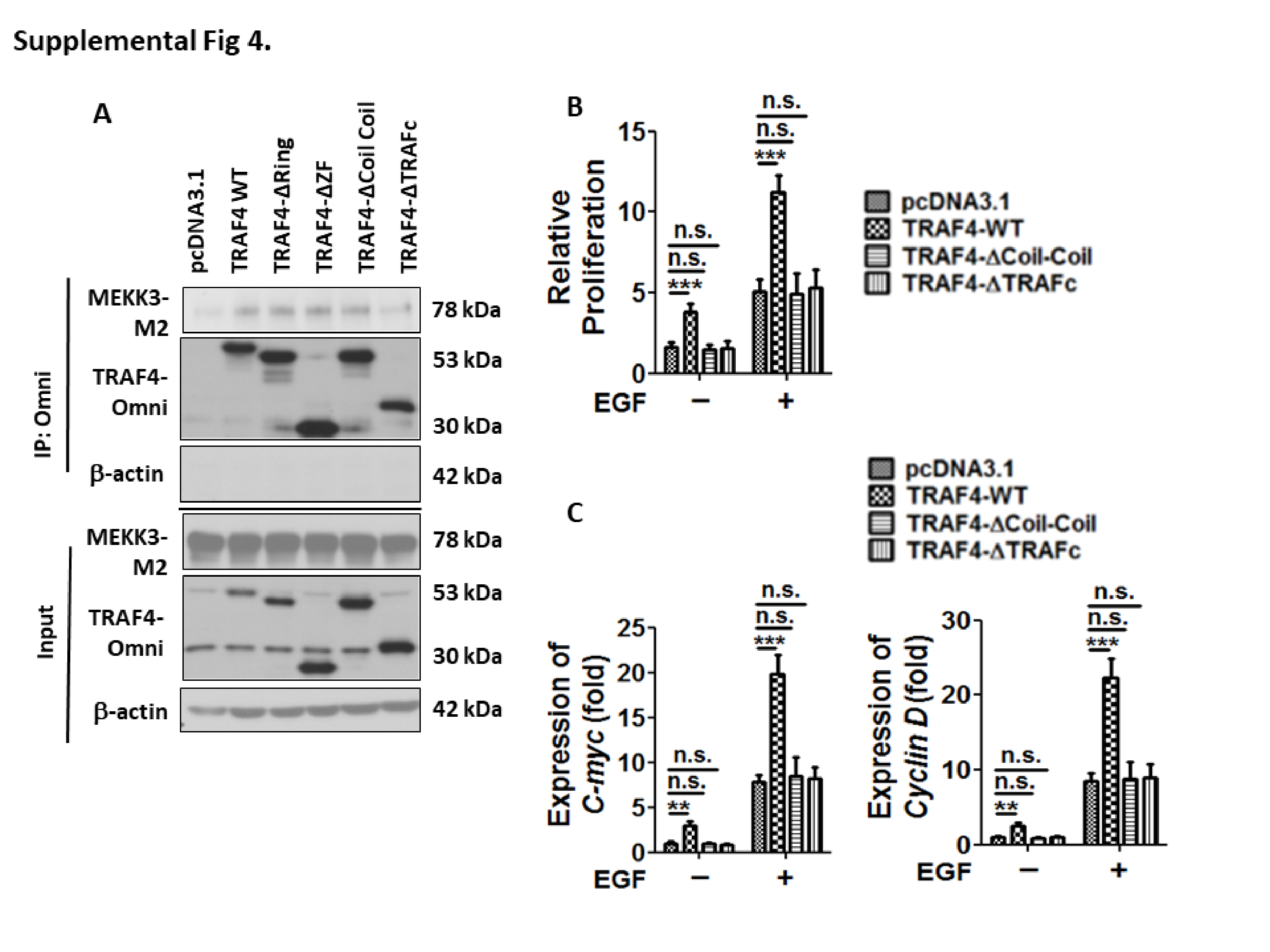

Supplement: Supplementary file 4 — Fig. S4. TRAF4 interacts with MEKK3 through the TRAFc domain. (A) TRAF4 KO cells were transfected with plasmids encoding MEKK3‐M2, as well as different domain‐deleted TRAF4‐Omni truncates. Cells were collected and lysed after 48 h of transfection for subjecting to immunoprecipitation with anti‐Omni antibody, followed by Western blot analysis. All the data are representative of three experiments. (B) TRAF4 KO A549 cells were transfected with plasmids encoding whole length TRAF4‐Omni, TRAF4 deltaTRAFc‐Omni or TRAF4 deltaCoil‐coil‐Omni. These cells were inoculated in 96 well plates at a density of 500/well in the presence or absence of EGF (1ng/mL). The cells proliferation was monitored by a CCK8 assay. Statistical significance was determined by a one‐way ANOVA. The error bars represented SEM. *** P < 0.001. (C) The relative expression level of Cyclin D and C‐myc mRNA was analyzed in cells as described in panel B. Statistical significance was determined by a one‐way ANOVA. The error bars represented SEM. * P < 0.05, ** P < 0.01, *** P < 0.001. The data is a representation of three independent experiments. [file FEB4-12-1747-s001.tif]

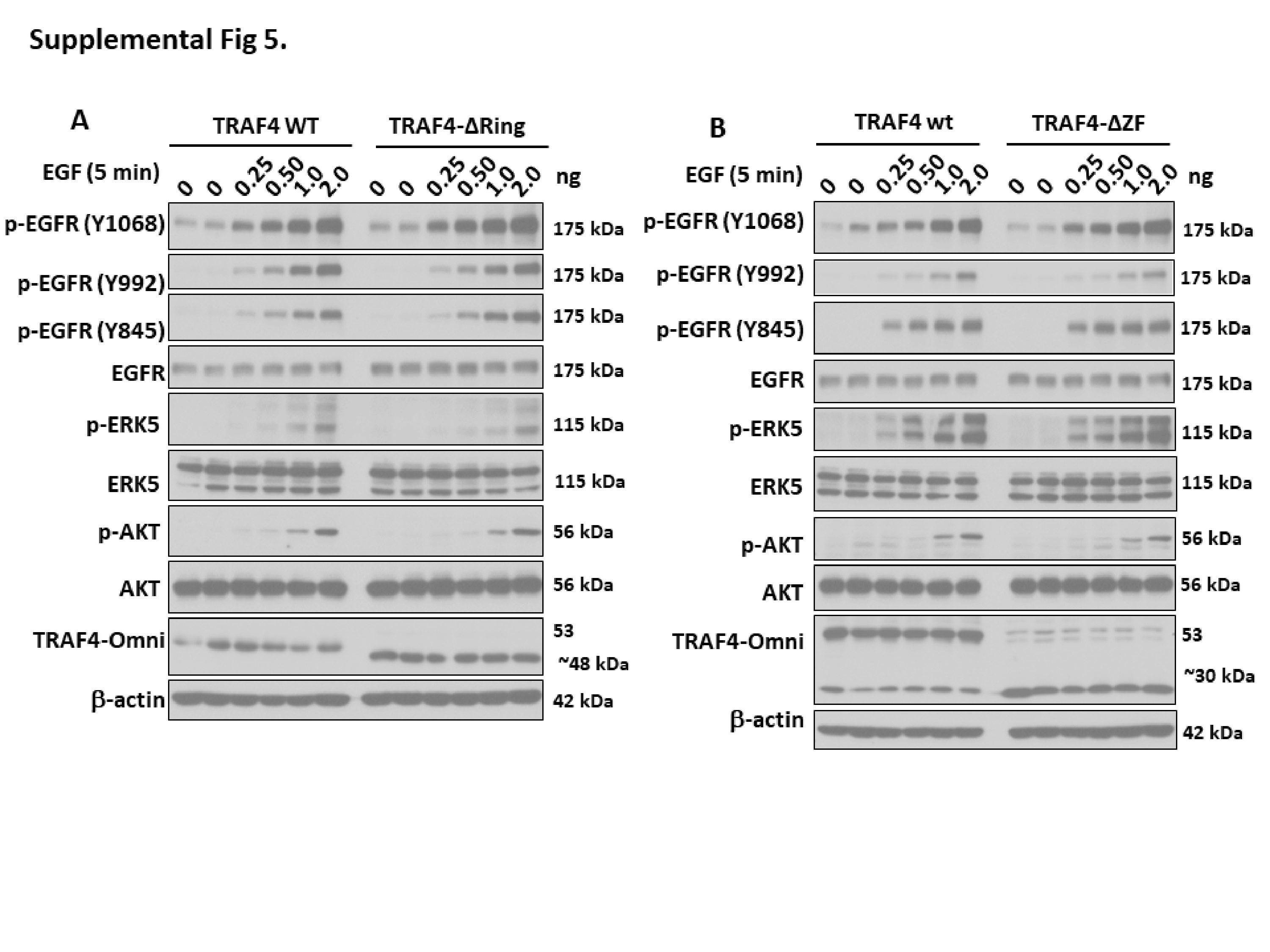

Supplement: Supplementary file 5 — Fig. S5. The role of Ring and ZF domain on TRAF4‐mediated EGFR/ERK5 activation. (A) TRAF4 KO A549 cells were transfected with plasmids encoding whole length TRAF4‐Omni or TRAF4 deltaRing‐Omni. (B) TRAF4 KO A549 cells were transfected with plasmids encoding whole length TRAF4‐Omni or TRAF4 deltaZF‐Omni. After 48 h of transfection, serum‐starved cells were stimulated by EGF for 5 min. Cell lysates were subjected to Western blot analysis using indicated antibodies. The data shown represent 3 or 4 independent experiments. [file FEB4-12-1747-s004.tif]
